# Evaluation of Antimicrobial Activity of Four Organic Acids Used in Chicks Feed to Control *Salmonella typhimurium*: Suggestion of Amendment in the Search Standard

**DOI:** 10.1155/2018/7352593

**Published:** 2018-10-01

**Authors:** Aicha El Baaboua, Mohamed El Maadoudi, Abdelhakim Bouyahya, Omar Belmehdi, Ayoub Kounnoun, Rajae Zahli, Jamal Abrini

**Affiliations:** ^1^Biology and Health Laboratory, Biotechnology and Applied Microbiology Team, Department of Biology, Faculty of Science, Abdelmalek Essaadi University, Tetouan, Morocco; ^2^Regional Laboratory for Analysis and Research, National Office for Food Safety, Tangier, Morocco; ^3^Laboratory of Human Pathology Biology, Faculty of Sciences, and Genomic Center of Human Pathology, Mohammed V University, Rabat, Morocco; ^4^Laboratory of Applied Biology and Pathology, Department of Biology, Faculty of Science, Abdelmalek Essaadi University, Tetouan, Morocco

## Abstract

Today, the general public has become increasingly aware of salmonellosis problems. Organic acids are known by their antimicrobial potential and commonly used for improving the quality of poultry feed. In this context, the present work evaluated the inhibitory effect of four organic acids, namely, acetic acid, citric acid, lactic acid, and tartaric acid, at different levels of contamination by *Salmonella typhimurium*. The neutralization of these organic acids *in vitro* and in the presence of one-day-old chick's organs was also investigated during the search for *Salmonella* serovars in birds as described in the Moroccan standard “NM 08.0.550.” The effect of four organic acids on *Salmonella typhimurium* was tested *in vitro* and in the presence of chick's organs at different concentrations set of strain and organic acids tested. The MIC results demonstrated that tartaric acid, citric acid, and acetic acid inhibited *Salmonella typhimurium* at concentrations of 0.312%, 0.625%, and 0.512% for the three levels of strain: 10, 100, and 10^3^ CFU/ml, respectively, while lactic acid and depending on the amount of the strain introduced acts differently: 0.078% for 10 CFU/ml and 0.156% for 100 and 10^3^ CFU/ml. The concentration of 0.04M of Na_2_HPO_4_ solution has proved, *in vitro*, in caecums and organs of chicks (in presence of organic acids) that strain introduced, even at low concentrations, can be recovered. The use of additives has beneficial effects in *Salmonella* control program. However, the present results recommend the amendment of *Salmonella* research standard, taking into account the probable presence of organic acids in digestive content of one-day-old chicks.

## 1. Introduction

Salmonellosis is a zoonotic disease that causes a serious issue in both human health and animal health. It is the major problem of morbidity and mortality that are regularly detected by official inspection authorities. The most reported serovars in *Salmonella* taxonomy are *Salmonella* Pullorum, *Salmonella* Gallinarum, and *Salmonella* Arizonae [1]. For instance, *Salmonella* is the second commonly reported bacteria causing gastrointestinal infection in the EU after *Campylobacter* [2, 3]. Each year, over 90,000 foodborne illness cases caused by *Salmonella* have been reported in the EU. This pathogen can have a large socioeconomic impact due to illness, medical costs, lost of productivity, disability, and deaths. In addition, the European Food Safety Authority (EFSA) has estimated that the overall economic burden of human salmonellosis could be as high as 3 billion dollars per year [4]. That is why *Salmonella* is an important target for the food industry, owing to their wide spread through international trade in animal feed, live animals, and food.

It is well documented that human *Salmonella* illnesses are caused by consumption of contaminated raw or undercooked food from animal origin, and it is known that the contamination process can happen during transportation, during packinghouse operations of meat products, or by cross contamination throughout processing steps such as evisceration or by slaughtering tools [5, 6]. In fact, chicken skin was reported to be an essential part of contamination and the most difficult site to control *Salmonella* [7, 8]. Many authors agree that animal feed is the first step to prevent contamination from the entrance, particularly in poultry [9–11].

Organic acids are designated and approved by the Federal Drug Administration (FDA) as safe substances (GRAS). Citric, lactic, tartaric, and acetic acids are the most known organic acids used, and their mechanism of action is previously mentioned [12–15]. They have been proved as effective sanitizers in reducing the bacterial charge (antimicrobial capacity and preservation of product quality), cost-effectiveness, and their simplicity. Indeed, the uses of these solvents differ from country to another depending on differences in legislation. Therefore, the use of organic acids is one of the most old and popular in decontamination procedures worldwide.

In Morocco, population growth rate has been trending up in last decade [16] and chicken meat is still the highest meat consumed in poultry sector because the prices are much cheaper than those of red meats or seafood [17, 18].

For this reasons, Morocco imports, from Spain and France, a considerable amount of birds to satisfy the population needs. The objective of the present work was to study the inhibitory effect of four organic acids on *Salmonella typhimurium*, to evaluate the efficacy of an alternative neutralizer tested *in vitro* and on the digestive contents of chicks imported by Moroccan companies, and to suggest amendment in the Moroccan standard of *Salmonella* research in one-day-old chicks.

## 2. Materials and Methods

### 2.1. Bacterial Strain and Condition

A pellet of *Salmonella typhimurium* ATCC^®^ 14028™ was grown overnight in brain heart infusion broth (Biokar, Beauvais, France) at 37°C and isolated on trypticase soy agar (TSA) (Becton Dickson Microbiology Systems, Cockeysville, Md.) plates. The pure colonies were multiplied on TSA and stored in physiological solution with 25% glycerol at −80°C until further use. The bacterial strain was enumerated using standard serial 10-fold dilutions in physiological saline solution.

### 2.2. Minimal Inhibitory Concentrations (MIC) and Minimal Bactericides Concentrations (MBC) of Organic Acids

The choice of organic acids is based on the list of food additives authorized for use in animal feed. The four acids used have a respective purity of 99.8% acetic acid, 99.5% tartaric acid, 99% citric acid, and 90% lactic acid. The MIC is studied at three different concentrations of the test strain: 10, 10^2^, and 10^3^ CFU/ml. To achieve the dilution of the organic acids, we need three sets of test tubes. Each set consists of 8 test tubes containing 9 ml of buffered peptone water (BPW). All organic acids tested in this study were diluted at a concentration of 5%. The measurements used for each acid were as follows: 0.5 ml of acetic acid in 10 ml of BPW, 0.56 ml of lactic acid (90%) in 10 ml of BPW, 0.5 g of tartaric acid in 10 ml of BPW, and 0.5 g of citric acid in 10 ml of BPW.

Nine milliliters of the organic acids stock solution was taken, and the dilutions were carried out at a half dilution in each case, so as to obtain the corresponding concentrations ranging from 2.5% to 0.0198%. In each series, the same dilution protocol of stock solution was carried out. Only strain concentration inoculated in each series has been changed. The tubes were incubated at 37°C for 18 to 24 h.

The study of the MBC was thus made, in our case, from the tubes used for the determination of the MIC and incubated for 18 to 24 h at 37°C. For each of the three levels of inoculums tested, and each of the organic acids tested, 1 ml of each tube was taken and placed in a Petri dish, followed by about 15 ml of TSA. The mixture was homogenized by orbital shaking by hand. The cultures were incubated at 37°C for 18 to 24 hours and colonies were counted the next day.

### 2.3. Neutralization of Inhibitory Effect of Acids by Using Universal Neutralizer

The choice of the neutralization solution was based on a universal neutral recommended in the standards by studying the bactericidal activity of disinfectants and antiseptics and also by the norm concerning the search for *Salmonella* in animal production environment “NM 08.0.549.”

The protocol consists in measuring the MIC of the studied acids in the presence of the neutralizing solution. The test was carried out in three successive steps:

*Dilution of Acid*. A half dilution of the acid is made by taking 8 ml of the 5% preparation which was added to the first tube set containing 8 ml of BPW for obtaining 2.5% acid. The serial acid dilution was prepared until the last test concentration.

*Addition of Neutralizer*. 1 ml of neutralizer stock solution was added to the series of organic acid dilution tubes.

*Addition of Strain*. After 5 to 10 min, the tubes of each organic acid dilution set tested were supplemented with 1 ml of one of the three bacterial suspensions prepared: 10^2^ CFU/ml, 10^3^ CFU/ml, and 10^4^ CFU/ml to each series, in order to obtain a final titer of 10 CFU/ml, 10^2^ CFU/ml, and 10^3^ CFU/ml, respectively.

### 2.4. Use of Buffer Solution Neutralizer

The choice of an alternative neutralizing solution has been taken into account, the fact that organic acids' action are mainly known by their capacity to acidify the medium matters that inhibit the bacterial growth. Starting from principle, neutralization of the acidity will make it possible to neutralize the inhibitory effect of the test dose under consideration. It was also the choice of a bearing on the components of the universal neutral, namely, the disodium hydrogen phosphate dodecahydrate (Na_2_HPO_4_). This solution was used at the same concentration in the universal buffer (0.01 M) = (1X concentrated solution) and at the concentrations 0.015 M, 0.02 M, and 0.04 M. This amounts to weighing, respectively, 17.14 g, 25.71 g, 34.28 g, and 68.56 g of Na_2_HPO_4_ in 200 ml of distilled water. The dissolution of the crystals was completed with magnetic stirring on a hot plate at 50°C.

#### 2.4.1. pH Measurement of Organic Acids before and after Neutralization

Four sets of polypropylene 15 ml conical bottom tube containing 8 ml of BWP were prepared for each organic acid handled. Then, the same protocol of acid dilution was carried out to have the test doses: 2.5%, 1.25%, 0.625%, 0.312%, and 0.0098%. Using a pH meter, the pH of each dilution acid was measured. Subsequently, 1 ml of phosphate buffer concentration tested was added and the pH was measured after stirring.

#### 2.4.2. Determination of MIC of Organic Acids Tested after Neutralization

The same protocol was repeated for the study of the MIC of organic acids for *Salmonella typhimurium* in the presence of the greatest phosphate buffer concentration as neutralizing solution (0.04 M). The final test doses of the organic acids were as follows: 2.5%, 1.25%, 0.625%, and 0.312%. The three inoculum levels of the tested strain were again studied: 10, 10^2^, and 10^3^ CFU/ml. The aim was to demonstrate that the greatest neutralizing solution has no toxic effect on the *Salmonella* strain, and also to determine the largest dose of acid that can be neutralized under the test conditions.

### 2.5. Neutralization of Organic Acids in the Presence of Chick's Organ Extracts:

*Organ Extract Preparation.* After the euthanasia of eight one-day-old chicks, the chicks were undressed by removing the skin previously soaked by alcohol 70°. Then, openings were made with the scissors in the thorax to remove the lungs, liver, cecum, and yolk sacs as well. All organs were pooled in a 50 ml sterile flask and mixed until a homogenate was obtained. The choice of the doses tested of each organic acid was based on the results of their MIC in the presence of the neutralizing solution.

Three sets were prepared for each acid corresponding to the strain concentrations of 10, 10^2^, and 10^3^ CFU/ml. Each tube contains 8 ml of organic acid-BPW, 1 g of organ homogenate, and then 1 ml of the neutralizing solution and was allowed to react for 5 to 10 minutes. At the end, 1 ml of the tested bacterial suspension was added. After exposure, incubation was carried out at 37°C for 18 h to 24 h. After that, the search for *Salmonella* was done according to the protocol described in Moroccan standard “NM 08.0.550.”

In order to confirm colonies obtained on selective isolation media of *Salmonella,* hektoen and xylose lysine deoxycholate agar (XLD), two boxes containing characteristic colonies were chosen for each organic acid, on which 5 colonies were taken from each box to undergo differentiation test between *Salmonella* and *Proteus*: test of tryptophan deaminase.

### 2.6. Application of the Methodological Parameters Studied on Laboratory Samples

The *Salmonella* research was carried out on the samples of 10 batches of 1-day-old chicks, each batch was composed of 5 chicks, and each of them was separated from the ceca in 50 ml sterile vials. The neutralizing solution was added after BWP dilution and incubation as described in the standard. The same protocol described in the standard of *Salmonella* research in foods has been used for the detection of *Salmonella* in organ extracts, adding 1 ml the neutralization solution (0.04 M), before preenrichment incubation, in order to neutralize any organic acid compound.

## 3. Results

### 3.1. MIC and MBC of Organic Acids

Table 1 shows the results obtained for the inoculum suspension count in each case of the four organic acids and the results of the MIC and the MBC studied at the different inocula. For acetic acid, the MIC was the same for the three levels of inoculums and it was equal to 0.312%, while the MBC determination shows that the first two inocula of 10 and 10^2^ CFU/ml have an equal value to MIC (0.312%). Contrary to the result obtained for the inoculum of 10^3^ CFU/ml, an increase in MBC was observed (0.625%).

In the case of tartaric acid, from these results, it appears that the strain tested has a MIC of 0.312% for the three levels of inoculums tested. The study of MBC of tartaric acid indicates that it has a similar value to MIC (0.312%) for the inoculum of 10 CFU/ml. On the other hand, the inoculums of 10^2^ and 10^3^ CFU/ml demonstrate the bactericidal action at a concentration of 0.625%.

For citric acid, as the table shows, the MIC was 0.625% for inoculums of 10, 10^2^, and 10^3^ CFU/ml. In addition, the bactericidal action was obtained only at a concentration of 1.25% for the three levels of inoculum.

Lactic acid, compared to the other three acids studied, has a very strong inhibitory action. At 10 CFU/ml, the inhibition of *Salmonella* occurred at a concentration of 0.078%. However, for the two inoculums of 10^2^ and 10^3^ CFU/ml, a MIC was obtained at 0.156%. Furthermore, the study of the MBC of the three series of inoculum showed that the bactericidal action was achieved at the same concentration (0.156%).

### 3.2. Neutralization of Organic Acids Inhibitory Effect by Using Universal Neutralizer

By using universal neutralizer, it was possible to neutralize, partially, the organic acids selected for this study.  Table 2 summarizes the results obtained from enumeration and the MIC study of the organic acids studied by using the universal neutralizer at the three levels of inocula studied.

It emerges that the neutralization of acetic acid was not effective, and the MIC was found to remain the same as before the addition of the neutralizer (0.312%).

The inhibitory action of tartaric acid was conditioned by the titer of the studied strain. It could be seen that for the inoculums of 10 and 10^2^ CFU/ml using the neutralizer, the MIC was 0.625%. It was also noted that, for 10^3^ CFU/ml, the MIC increases from 0.312% before neutralization to 1.25% after the addition of neutralizer.

In citric acid, by resemblance to tartaric acid, the titer of inoculum introduced plays a decisive factor in the MIC. At 10 CFU/ml, the MIC for citric acid was 0.625, while at 10^2^ and 10^3^ CFU/ml, the MIC was the same as that present in tartaric acid at the same inoculum (1.25%). However, the MIC of lactic acid in the presence of neutralizer is 0.625% for the three inoculums introduced.

**Figure 1:**
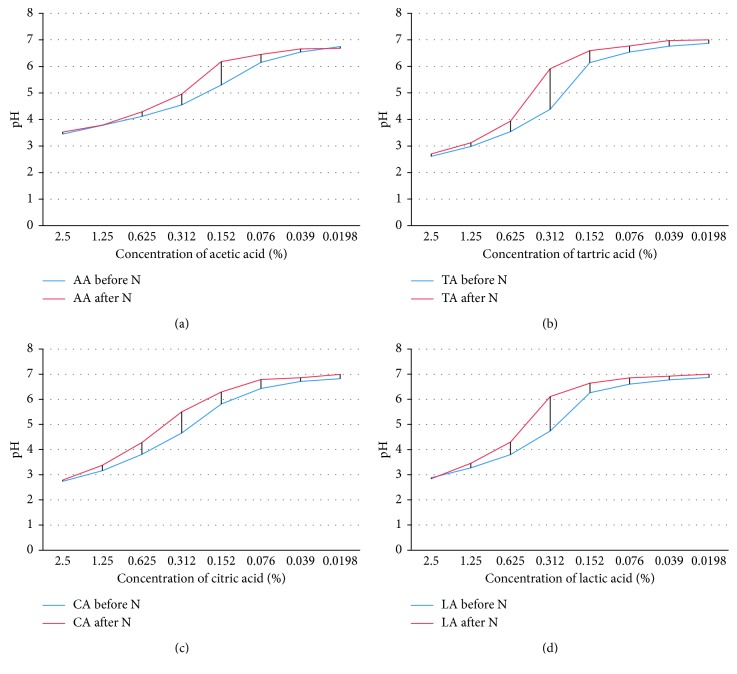
pH measurement curves of the four organic acids' concentrations using universal neutralizer.

The results of the pH measurements of the four organic acids before and after the neutralization were collected and are presented in Figure 1. These results show that, before neutralization, the pH was clearly acidic at the first test doses used and the values obtained increased exponentially with dilution of acids to reach neutrality at the last two test doses studied. Generally, the pH values obtained were overall less than or equal to 5 for the test doses of 2.5 to 0.312% and greater than or equal to 5 for the other doses.

After neutralization, the values recorded were less than or equal to 5 for only the first three test doses (2.5% to 0.625%), in the case of citric, lactic, and tartaric acids, whereas these values were greater than or equal to 6 for the other doses (0.312% to 0.0198%). Acetic acid showed resistance to neutralization with pH ≤ 5 for doses of 2.5 to 0.312% and pH ≥ 6 for doses of 0.152 to 0.0198%.

### 3.3. Use of Buffer Solution Neutralizer

#### 3.3.1. pH Measurement

**Figure 2:**
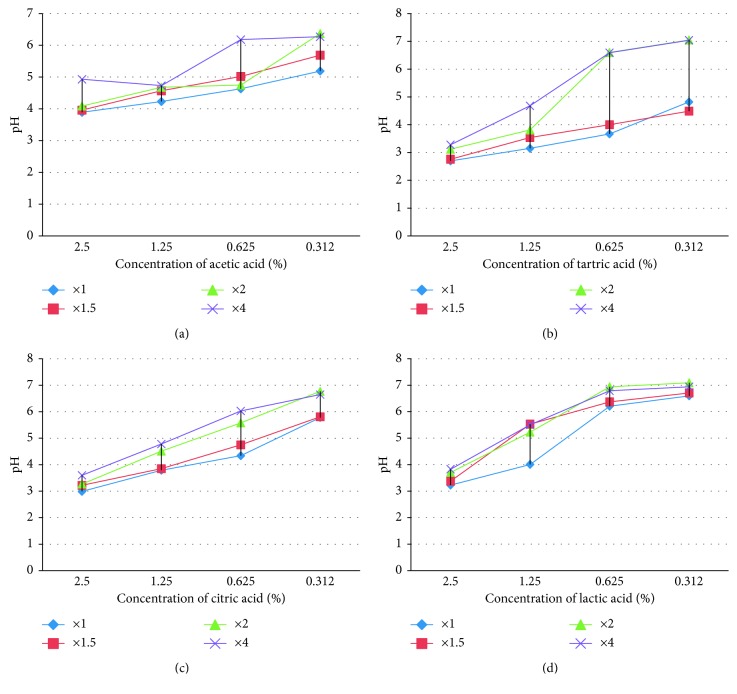
pH measurement curves of organic acids studied at different concentrations of the disodium hydrogen phosphate dodecahydrate (Na_2_HPO_4_).

The results of pH measurements of the four organic acids after neutralization, using disodium phosphate buffer solution at four different concentration levels: 0.01 M, 0.015 M, 0.02 M, and 0.04 M are summarized in Figure 2. It emerges that the results for the concentration of the 0.01 M buffer solution, the concentration of the universal neutralizer, are the same as when using this latter.

In the case of acetic acid, the neutralization (pH ≥ 6) is obtained with the 0.02 M and 0.04 M of solution from the concentrations of 0.312% and 0.625%, respectively.

For tartaric and citric acids, neutralization by 0.02 M and 0.04 M solutions makes it possible to have pH values ≥ 5 from 0.625%. A pH ≈ 5 is also obtained for 1.25% for acetic, tartaric, and citric acids after neutralization by 0.04 M of buffer solution.

In the case of lactic acid, the neutralization appears to be easier, pH values ≥ 6, by using the buffer solutions from 0.625%. The values of pH ≥ 5 are also obtained at a dose of 1.25% by 0.015 M, 0.02 M, and 0.04 M of buffer solutions.

#### 3.3.2. Organic Acids Neutralization Test

From the results obtained (Table 3), it is clear that neutralizing solution at 0.04 M did not interfere with *Salmonella* culture and the three organic acids, tartaric, citric, and lactic acids, could be neutralized up to a concentration of 1.25% for the three inoculums tested, whereas for acetic acid, this neutralization did not occur. It is obtained only for maximum concentrations of 0.32% for the three inocula tested and 0.625% for inoculums of 10^2^ and 10^3^ CFU/ml.

### 3.4. Study of Inhibitory Effect and Neutralization Test of Organic Acids in the Presence of Organ Extracts

For tartaric acid, the tested strain was reisolated after exposure to the three doses tested: 2.5, 1.25, and 0.625% of acid, using the two enrichment (Selenite Cystine broth and MRSV) and isolation media used (XLD and Hektoen).

In the enrichment step on Selenite Cystine broth, the strain was recovered in citric acid case at a dose of 2.5% only for an inoculum of 103 CFU/ml and also at a dose of 1.25% for inocula of 102 and 103 CFU/ml. While it was recovered for the three inocula tested at the dose of 0.625%. In addition, it was very interesting to note that the MRVS medium makes it possible to scour the strain, at all doses and for the three inocula.

For lactic acid, the strain was reisolated at a dose of only 2.5% for the inoculum of 10^3^ CFU/ml after enrichment and isolation. However, it was recovered at test doses of 0.625% and 1.25% for the three inocula in all isolation media.

In the case of acetic acid, the reisolation of *Salmonella typhimurium* was obtained just at the test doses of 0.312% and 0.625% for the three levels in all media used.

It should be noted that the confirmation of the reisolation of *Salmonella typhimurium* was done during the neutralization tests in the presence of organ extracts, by carrying out the phenylalanine deaminase test on the characteristic colonies of *Salmonella* in order to differentiate them from *Proteus* sp. 10 colonies of isolates made for each acid were 100% negative for the test performed. Therefore, all colonies tested were *Salmonella* strains.

### 3.5. Application of Methodological Parameters Studied in *Salmonella* Detection on Laboratory Samples

After preenrichment incubation, the samples enriched in Selenite Cystine broth and modified semisolid Rapport-Vassiliadis (MRVS) and isolated on Hektoen and XLD medium showed that all tested samples were negative (absence of *Salmonella* characteristic colonies) even after further incubation (up to 48 h) of isolation media.

## 4. Discussion

Organic acids offer several advantages as antimicrobials molecule, because of their low cost and the easy manipulation [11]. The early use of organic acids in the production chain helps to prevent the spread of *Salmonella* contamination in all production devices as well as during feedstuff storage [19].

Significant differences in the effects of acids on reduction of *Salmonella* in poultry feed have been observed [13, 20, 21]. The results of the MIC and MBC study were compared to the other three acids studied; lactic acid has the strongest inhibitory action on the *Salmonella typhimurium* strain. The inhibitory dose for the three levels of inoculum was 0.156%, while citric acid had a lower action, with an increased MIC at different levels of inoculums (0.625%). Tartaric acid, as well as acetic acid, had a mean action with a MIC of 0.312% for the three levels of inocula tested. These results were in agreement with the results obtained in a research work entitled the use of acetic acid and citric acid to control of *Salmonella typhimurium* in tahini [22]. This can be explained by the pH-acid inhibitor of *Salmonella*, produced by these organic acids as demonstrated in pH results obtained from the acids solutions and their neutralization (Figure 1). Moreover, the efficacy differences of organic acids against *Salmonella* have been determined by various factors, such as the nature and physical form of the preparation and composition of the treated feed [11, 12].

For the neutralization of the inhibitory effects of acids, it should be noted that the universal neutralizer has a rather limited efficiency, since its neutralizing effect is obtained for lactic, tartaric, and citric acids only at a dose of 0.312% for the three levels of inoculums tested, whereas it is completely ineffective towards the citric acid (MIC), thing that makes use of this neutralizer for the recovery of *Salmonella typhimurium* exposed to this acid was critical, especially for acetic acid. When verifying the pH of test doses of solutions before and after the addition of the universal neutralizer, we found that, before neutralization, the pH values obtained were less than or equal to 5 for the test doses of 2.5 to 0.312% and greater than or equal to 5 for other doses, with an absence of bacterial growth in the first case and its presence in the second. As a result, the inhibition of bacterial culture coincides with pH values ≤ 5. This is all the more true as the neutralization of the inhibitory effect is accompanied by an increase in pH values, which testifies the close relationship between the decrease in pH and the inhibition of bacterial growth described in the literature [11, 12].

These results could be confirmed when using the alternative neutralizing solutions prepared with Na_2_HPO_4_, one of the main components of the universal neutralizer, with different molarities. For 0.01 M buffer solution concentration, the concentration of the universal neutralizer, the results were the same as when using the latter. This goes in the direction of the affirmation that the inhibitory action of these organic acids is mainly through acidification of the medium, which leads to a stop of multiplication or even a bacterial mortality. For 0.04 M concentration of the neutralizing solution, the three tartaric, citric, and lactic acids could be neutralized to a concentration of 1.25% for the three inocula tested. For acetic acid, this neutralization is obtained for the maximum concentrations of 0.32% for the three inocula tested and 0.625% for inoculums of 10^2^ and 10^3^ CFU/ml. Neutralizing solution at 0.04 M has no toxic effect on the *Salmonella typhimurium*. These results are complemented by those of the study of the inhibitory effect and the neutralization test of organic acids in the presence of organ extracts that show the capability of recuperating the strain at test doses of 0.625% and 1.25% for the three inoculums and in different media for lactic acid.

The strain was recovered from the organ extracts, in the case of enrichment on Selenite Cystine broth, for the three inocula tested at a dose of 0.625%. In addition, it was very interesting to note the high sensitivity of Selenite broth to the detection of small numbers of *Salmonella* [23–25]. The test strain was reisolated after exposure to the three doses tested: 2.5, 1.25, and 0.625% of acid on the enrichment media (Selenite broth and MRVS) and on XLD and Hektoen. The reisolation of the strain of organ extracts was obtained at a dose of 0.625% for the three levels of inoculums. Despite the fact that, during neutralization in the absence of the organ extracts, the 0.04 M neutralizing solution was not able to recover the strain at an inoculum of 10 CFU/ml. This could be explained by the technique used in this study, after neutralization, cannot recover the bacterial cells injured by organic acids. This makes it possible to amplify the neutralization of organic acids (in the case of acetic acid at a dose of 0.625% for the inoculum of 10 CFU/ml).

To our knowledge, the present work is the first study about the neutralization of organic acid in chick's organs. The results obtained allow us to propose an amendment to *Salmonella* search method “NM 08.0.550” in the chick used by the Moroccan surveillance laboratory (taking into account the possible presence of authorized organic acids). This amendment consists of proceeding in parallel to the standardized method and in the same way by preenrichment of the samples analyzed, supplemented by 1 ml of 0.04 M disodium phosphate buffer neutralizer. Thus, about 10 samples of imported chicks were tested for *Salmonella* by this method, in parallel to the official control carried out by the laboratory, and were found negative.

## 5. Conclusion

*Salmonella* is the leading pathogen responsible for food poisoning worldwide and causes an economic burden, through both healthcare use and lost productivity. The Moroccan standard of *Salmonella* research based on three steps (preenrichment, enrichment, and isolation) remains the method of choice used in national programs for surveillance and control of salmonellosis. Nowadays, an impressive range of organic acids is used in poultry feed to reduce *Salmonella* number and other bacteria in digestive tract of animals. The intensive use of organic acids can interfere with the *Salmonella* detection method by inhibiting their multiplication during the preenrichment and enrichment steps without eliminating the bacteria that could be an attack for *Salmonella* control program.

Our work has clearly established that the four organic acids tested interfered effectively with the *Salmonella* search standard from 0.078 to 0.312% of acid, but also depending on acid nature. Based on the results, it was concluded that the use of a universal neutralizer as stipulated in the *Salmonella* standard is not effective in neutralization of the organic acids tested. Moreover, this interference can be controlled by making an amendment to the standardized method, by introducing a neutralization step, at the time of preenrichment in peptone water (the addition of 1 ml of neutralizer based on 0.04 M disodium phosphate buffer). Consequently, the *Salmonella* methods will be optimized to detect the minimum level of contamination in chick's samples.

## Figures and Tables

**Table 1:** MIC and MBC enumeration of the four organic acids tested on *Salmonella typhimurium* ATCC^®^ 14028™.

	Strain titer (CFU/ml)	SC (CFU/ml)	Test doses of organic acids (%)							
			2.5	1.25	0.625	0.312	0.156	0.078	0.039	0.0195
Acetic acid	10	8	−−− (0)	−−− (0)	−−− (0)	−−− (0)	+++ (>300)	+++ (>300)	+++ (>300)	+++ (>300)
	10^2^	27	−−− (0)	−−− (0)	−−− (0)	−−− (22)	+++ (>300)	+++ (>300)	+++ (>300)	+++ (>300)
	10^3^	243	−−− (0)	−−− (0)	−−− (104)	−−− (>300)	+++ (>300)	+++ (>300)	+++ (>300)	+++ (>300)

Citric acid	10	9	−−− (0)	−−− (0)	−−− (31)	+++ (>300)	+++ (>300)	+++ (>300)	+++ (>300)	+++ (>300)
	10^2^	133	−−− (0)	−−− (0)	−−− (>300)	+++ (>300)	+++ (>300)	+++ (>300)	+++ (>300)	+++ (>300)
	10^3^	>300	−−− (0)	−−− (0)	−−− (>300)	+++ (>300)	+++ (>300)	+++ (>300)	+++ (>300)	+++ (>300)

Tartaric acid	10	28	−−− (0)	−−− (0)	−−− (0)	−−− (6)	+++ (>300)	+++ (>300)	+++ (>300)	+++ (>300)
	10^2^	114	−−− (0)	−−− (0)	−−− (4)	−−− (>300)	+++ (>300)	+++ (>300)	+++ (>300)	+++ (>300)
	10^3^	>300	−−− (0)	−−− (0)	−−− (1)	−−− (>300)	+++ (>300)	+++ (>300)	+++ (>300)	+++ (>300)

Lactic acid	10	17	−−− (0)	−−− (0)	−−− (0)	−−− (0)	−−− (0)	−−− (>300)	+++ (>300)	+++ (>300)
	10^2^	185	−−− (0)	−−− (0)	−−− (0)	−−− (0)	−−− (0)	+++ (>300)	+++ (>300)	+++ (>300)
	10^3^	>300	−−− (0)	−−− (0)	−−− (0)	−−− (0)	−−− (0)	+++ (>300)	+++ (>300)	+++ (>300)

−−−: absence of strain, +++: presence of strain; SC: strain control.

**Table 2:** MIC of organic acids in the presence of neutralizer for inocula of 10, 10^2^, and 10^3^ of *S. typhimurium*.

	Strain titer (CFU/ml)	SC (CFU/ml)	Test doses of organic acids (%)							
			2.5	1.25	0.625	0.312	0.156	0.078	0.039	0.0195
Acetic acid	10	19	−−−	−−−	−−−	−−−	+++	+++	+++	+++
	10^2^	220								
	10^3^	>300								

Citric acid	10	25	−−−	−−−	−−− +++	+++	+++	+++	+++	+++
	10^2^	286								
	10^3^	>300								

Tartaric acid	10	17	−−−	−−−	−−− +++	+++	+++	+++	+++	+++
	10^2^	263								
	10^3^	>300								

Lactic acid	10	34	−−−	−−−	−−−	+++	+++	+++	+++	+++
	10^2^	286								
	10^3^	>300								

−−−: absence of *Salmonella typhimurium*, +++: presence of *Salmonella typhimurium*, SC: strain control.

**Table 3:** MIC of the organic acids using 0.04 M of Na_2_HPO_4_ solution, for inoculums of 10, 10^2^, and 10^3^ of tested strain.

	Inoculum (CFU/ml)	Test doses of organic acids			
		2.5%	1.25%	0.625%	0.312%
Tartaric acid	10	−−−	+++	+++	+++
	10^2^				
	10^3^				

Citric acid	10	−−−	+++	+++	+++
	10^2^				
	10^3^				

Lactic acid	10	−−−	+++	+++	+++
	10^2^				
	10^3^				
Acetic acid	10	−−−	−−−	−−− +++	+++
	10^2^				
	10^3^				

## Data Availability

The data used to support the findings of this study are included within the article.
